# ChatGPT delivers mostly satisfactory but occasionally inaccurate and potentially unsafe answers to hip arthroscopy questions during the surgeon's learning curve

**DOI:** 10.1002/jeo2.70770

**Published:** 2026-05-25

**Authors:** Ingo Jörg Banke, Stefan Fickert, Stefan Landgraeber, Gregor Möckel, Alexander Zimmerer, Nikolai Ramadanov, Timoty Osterberger, Vanessa Twardy, Alexander Gebhart, Florian Pouessel

**Affiliations:** ^1^ Clinic of Orthopaedics and Sports Orthopaedics, School of Medicine and Health TUM University Hospital, Technical University of Munich Munich Germany; ^2^ AGA ‐ Society for Arthroscopy and Joint Surgery, Hip Committee Zurich Switzerland; ^3^ Sporthopaedicum Straubing Straubing Germany; ^4^ Department of Orthopedics and Orthopedic Surgery Saarland University Medical Center Homburg Germany; ^5^ MVZ Orthopädie Karlshorst Berlin Germany; ^6^ Gelenk Quartier Karlsruhe‐Durlach Germany; ^7^ Center of Orthopaedics and Traumatology, Brandenburg Medical School University Hospital Brandenburg an der Havel Brandenburg an der Havel Germany; ^8^ Department of Orthopaedics and Traumatology Universitätsklinikum Wiener Neustadt Wiener Neustadt Austria

**Keywords:** artificial intelligence, ChatGPT, hip arthroscopy, large language models, surgical learning curve

## Abstract

**Purpose:**

To assess the accuracy, potential safety concerns and readability of single‐shot answers generated by the free GPT‐4o ChatGPT interface to 15 predefined surgeon‐level hip arthroscopy (HAS) learning‐curve questions, using expert ratings (Mika scale) and interrater reliability analysis.

**Methods:**

Fifteen questions were selected based on frequency in HAS teaching courses. Each question was submitted once to ChatGPT in a new chat without additional prompting. Eight high‐volume hip arthroscopists, serving as faculty and trainers, independently rated every answer using the 4‐point Mika scale (1 = excellent, 4 = unsatisfactory). Consensus ratings were defined by the modal score or, in case of ties, by panel discussion with safety‐oriented adjudication. Interrater reliability was evaluated using intraclass correlation coefficients (ICCs). Readability metrics were assessed using the Flesch Reading Ease Score (FRES) and the Flesch‐Kincaid Grade Level (FKGL).

**Results:**

After consensus, 2 of 15 responses (13.3%) were rated excellent, 9 (60%) satisfactory with minimal clarification required, 3 (20%) satisfactory with moderate clarification required and 1 (6.7%) unsatisfactory, yielding a mean accuracy score of 2.2 ± 0.8 (median, 2.0; range, 1–4). The single unsatisfactory answer addressed patient positioning, and pharmacologic venous thromboembolism prophylaxis was rated satisfactory but raised safety concerns. Interrater reliability was moderate for single ratings (ICC(2, 1) = 0.58) and excellent for the mean of all raters (ICC(2, 8) = 0.92). Readability indicated a college‐level demand (mean FRES 34, mean FKGL 13).

**Conclusions:**

GPT‐4o provided mostly satisfactory and useful answers to common HAS questions posed by surgeons in their learning curve, but a minority of responses required substantial clarification or were judged unsafe if applied uncritically. These findings support the use of large language models as an adjunct educational tool, while highlighting the need for expert verification in safety‐critical topics.

**Level of Evidence:**

Level IV, cross‐sectional, comparative simulation study.

**Abbreviations**
AI: artificial intelligence
CPM: continuous passive motion
CT: computed tomography
FAQs: frequently asked questions
FKGL: Flesch–Kincaid Grade Level
FRES: Flesch Reading Ease Score
GPT‐4: Generative Pre‐trained Transformer 4
HAS: hip arthroscopy
HO: heterotopic ossification
MRI: magnetic resonance imaging
Q&A: questions and answers
ROM: range of motion
THA: total hip arthroplasty

## INTRODUCTION

Hip arthroscopy (HAS) has a steep technical and cognitive learning curve [22]. Freely available large language models (LLMs) such as ChatGPT (Generative Pre‐trained Transformer; GPT), released 30 November 2022, are increasingly used informally by trainees as just‐in‐time educational tools. In parallel, artificial intelligence (AI) in orthopaedics is expanding rapidly, with a marked rise in publications, particularly in image interpretation, treatment planning and prognostic assessment [10, 11, 28]. ChatGPT and similar chatbots may support medical education (e.g., case simulations and procedural questions and answers [Q&A]) [4] and have shown potential in clinical decision support by generating assessments and treatment plans from clinical and imaging data, even in complex scenarios [4, 21]. However, these models require critical oversight due to limitations in data quality, interpretability and accountability [6, 13].

From a clinical perspective, this trend is particularly relevant in HAS because many decisions during the learning curve are technique‐dependent and safety‐critical (e.g., positioning including postless setup, traction application and monitoring [35], portal placement and trajectories relative to adjacent neurovascular structures [27], capsular management/closure strategy [30], particularly in instability‐risk morphologies and perioperative prophylaxis [17, 31]. In this setting, inaccuracies or overly confident generalizations may be applied inappropriately and could plausibly affect patient safety if used uncritically. As surgeons in the HAS learning phase increasingly consult LLMs, their performance on surgeon‐level, safety‐critical questions remains uncertain, given that such information may be applied directly to patient care.

Prior studies in HAS have assessed ChatGPT exclusively using patient‐focused frequently asked questions (FAQs), which likely carry lower expectations and lower technical complexity [1, 2, 3, 9, 15, 16, 26, 33]. Overall, responses were rated satisfactory to excellent, but occasional inaccuracies and high reading complexity (college‐level Flesch Reading Ease Score [FRES]/Flesch‐Kincaid Grade Level [FKGL]) were reported. Similar findings have been described for patient‐facing total hip arthroplasty (THA) questions, with mostly satisfactory or excellent answers but occasional need for clarification [23]. In contrast, early surgeon‐focused evaluations in knee and shoulder topics suggest more variable quality, combining helpful content with relevant gaps in detail and safety [7, 25]. Targeted work on common meniscus surgery questions likewise reports generally satisfactory answers but inconsistent depth and some deficiencies [36].

From a clinical education perspective, HAS decisions during the learning curve are anatomy‐ and technique‐dependent and may directly influence intraoperative choices. Therefore, evaluating LLM performance on surgeon‐level, safety‐critical learning‐curve questions addresses an unmet gap not captured by patient‐facing FAQ assessments. Despite these patient‐focused, hip‐related ChatGPT studies, the quality and safety of responses to surgeon‐level, technically detailed HAS questions have not yet been evaluated. During the learning phase, HAS surgeons require nuanced, technique‐based and safety‐critical information, often directly relevant to intraoperative decision‐making. It is unknown whether ChatGPT's free GPT‐4o web interface meets this higher bar for accuracy, completeness and safety when addressing surgeon questions. Accordingly, a structured evaluation of surgeon‐level learning‐curve questions may help delineate where LLM outputs are generally reliable for first‐pass orientation versus where expert verification should be mandatory to mitigate safety risks.

Mika et al. proposed a 4‐point rating system to categorize ChatGPT response quality based on completeness and the need for clarification, and it has been applied across multiple orthopaedic topics, including THA, HAS and meniscus surgery [1, 23, 26, 33, 36]. Readability is another relevant dimension, as prior studies have shown that ChatGPT‐generated medical responses often exceed recommended reading levels (FRES/FKGL) [2, 15], which may limit efficient uptake in time‐pressured learning‐curve settings.

The aim of this study was to evaluate the accuracy and potential safety concerns of answers generated by the free GPT‐4o ChatGPT web interface to common surgeon‐level HAS learning‐curve questions, and to assess interrater reliability and readability. We hypothesized that most responses would be rated satisfactory (Mika 1–2), but that a relevant minority, particularly on safety‐critical topics such as patient positioning, traction, capsular management and thromboprophylaxis, would require substantial clarification or be judged potentially unsafe if applied uncritically, underscoring the need for expert verification during training.

## METHODS

### Study design

This prospective observational content analysis evaluated the accuracy, clarity and educational usefulness of ChatGPT‐4o responses to surgeon‐level questions commonly encountered during the HAS learning curve. The methodology followed established standards from prior orthopaedic LLM research and reporting practices [2, 19, 23], and comprised three phases: (1) systematic identification of high‐frequency trainee questions, (2) standardized acquisition of ChatGPT‐4o responses and (3) structured expert evaluation using a validated rating system. No patient participation, interventions, clinical records or identifiable individual‐level data were involved. Therefore, institutional review board approval was not required. The study was conducted in accordance with the Declaration of Helsinki and ethical principles for educational research and scholarly transparency.

### Question source

Common HAS questions typically raised by surgeons during the learning curve in HAS instructional courses and observerships held under the auspices of Europe's largest arthroscopy society (AGA ‐ Society for Arthroscopy and Joint‐Surgery) were contributed by eight board‐certified, fellowship‐trained orthopaedic surgeons serving as active HAS teaching faculty (I.J.B., S.F., S.L., G.M., A.Z., V.T., A.G. and F.P.). To contextualize the learning‐curve setting from which these questions arose, typical trainee lifetime HAS exposure was estimated for both assistant and primary surgeon roles, corresponding predominantly to 21–50 assisted cases and 1–20 performed cases, respectively. Accordingly, the questions analysed in this study reflect the typical participant profile of our HAS instructional courses over recent years and an early learning‐curve stage in which surgeons may seek additional decision support, including LLMs. Recurring questions were compiled into an initial dataset and then reviewed and iteratively consolidated to yield 15 unique questions reflecting the most common educational needs of early‐career hip arthroscopists (Table 1).

**Table 1:** The 15 most frequently asked learning‐curve questions in HAS.

	Questions
1	Which types of hip arthroscopy cases are most appropriate for surgeons early in the learning curve?
2	What preoperative imaging is recommended before hip arthroscopy?
3	What patient positioning is recommended for surgeons early in the hip arthroscopy learning curve?
4	What standard portal placement is recommended for hip arthroscopy?
5	How long is traction safe during hip arthroscopy?
6	For femoroacetabular impingement syndrome, which capsular management is generally recommended in hip arthroscopy?
7	In hip arthroscopy, when is labral repair preferred over debridement?
8	During hip arthroscopy, how can adequacy of cam resection be assessed?
9	During hip arthroscopy, how can adequacy of pincer resection be assessed?
10	What intraoperative complications are most common during hip arthroscopy?
11	After hip arthroscopy, what postoperative rehabilitation protocol is recommended?
12	After hip arthroscopy, what prophylaxis is recommended against heterotopic ossification?
13	After hip arthroscopy, what venous thromboembolism prophylaxis is recommended?
14	In hip arthroscopy, how many cases are typically required to overcome the main learning curve?
15	Which patient factors predict poor outcomes after hip arthroscopy?

Abbreviation: HAS, hip arthroscopy.

### ChatGPT model and query protocol

All answers were generated using the latest free web‐based ChatGPT interface (GPT‐4o; OpenAI) with default settings (no paid features, beta functions or customization). On 14 December 2025, each question was submitted once in a new, independent chat session to prevent contextual carryover, consistent with prior LLM evaluations [19, 23]. A single standardized prompt format was used, with no follow‐up prompts, clarifications, hints or reformulations. This single‐shot approach was intended to mirror real‐world use by surgeons in training. All queries were submitted in English, and each unedited response was copied verbatim into a data sheet by two study team members who were not involved in question sourcing or expert rating (N.R. and T.O.).

### Expert raters

Expert assessment was performed by eight board‐certified, fellowship‐trained senior orthopaedic surgeons with high‐volume HAS practices (I.J.B., S.F., S.L., G.M., A.Z., C.F., O.S. and W.S.). Each rater performs approximately 200–400 HAS procedures per year, with an estimated lifetime volume of 5000–15,000 procedures, and is actively involved in HAS teaching and training. Raters were not involved in querying the model and were provided only the verbatim ChatGPT‐4o outputs for evaluation. Responses were presented in a randomized order for each rater (computer‐generated randomization). Each rater independently evaluated all 15 responses and was blinded to the other raters' evaluations and to the final consensus ratings during initial scoring.

### Rating system and consensus definition

Responses were evaluated using the four‐point accuracy and clarity scale described by Mika et al. (Supporting Information S1: Supplement 1), which has been used in prior orthopaedic LLM research [23]. Ratings were assigned as follows:

1. Excellent—Fully accurate, consistent with contemporary practice, sufficiently detailed; no clarification required.
2. Satisfactory, minimal clarification—Fundamentally accurate and evidence‐based; minor additions or clarifications would improve completeness.
3. Satisfactory, moderate clarification—Partial or outdated/redundant information; important clarifications or additions are needed; key aspects may be missing.
4. Unsatisfactory—Incorrect, misleading or overly generalized information such that misinterpretation or patient harm could realistically occur.

In addition to numerical ratings, experts provided brief narrative comments on accuracy, missing content, conceptual coherence and potential safety concerns. For each question, the consensus rating was defined as the modal score across the eight raters. In the absence of a clear mode, ratings were discussed to reach consensus; if disagreement persisted, a safety‐oriented adjudication was applied, and the less favourable category was assigned.

### Outcomes

The primary outcome was response quality for each question, quantified by the expert accuracy rating using the Mika score (1–4) (consensus rating and rater means). Secondary outcomes were (1) interrater reliability among experts (ICC for single measures and mean measures) and (2) readability of generated responses, assessed by FRES (0–100) and FKGL, consistent with prior patient‐focused HAS LLM evaluations [2, 15]. Higher FRES indicates easier readability, whereas FKGL estimates the U.S. grade level required for comprehension. Scores were calculated from the unedited ChatGPT‐4o output using standard formulas and are reported as mean ± standard deviation and range. Exploratory analyses compared readability between higher‐rated responses (Mika 1–2) and those requiring more substantial clarification (Mika 3–4).

### Statistical analysis

Descriptive statistics were calculated for the primary outcome (distribution of Mika categories, mean ± standard deviation [SD], median, range). Interrater agreement (secondary outcome) was assessed using a two‐way random‐effects intraclass correlation coefficient (ICC) for absolute agreement (single and mean measures). Readability metrics (secondary outcome) were summarized as mean ± SD and range, and exploratory comparisons were performed between higher‐rated (Mika 1–2) and lower‐rated (Mika 3–4) responses. Exploratory analyses examined associations between readability metrics and accuracy ratings and compared readability between higher‐rated responses (Mika 1–2) and those requiring more substantial clarification (Mika 3–4) using nonparametric tests. Analyses were performed using R (Version 4.2.2). Given the descriptive nature of the study, emphasis was placed on distributional patterns and effect magnitudes rather than formal hypothesis testing. In accordance with previously published descriptive studies, no a priori power calculation for hypothesis testing was performed; however, a reliability‐based sample size justification was applied. The study was originally planned with five raters, and sample size (*n* ≈ 15 items) was chosen to reflect frequent course‐derived questions and to meet commonly used ICC sample‐size recommendations (target ICC ≥ 0.70 vs. minimally acceptable ICC ≤ 0.40, *α* = 0.05, 80% power). Subsequently, the rater pool was increased to 8 experts, which was expected to further improve the precision of ICC estimates. This was supported by the observed interrater agreement (i.e., improved mean‐measures reliability with the larger rater panel).

## RESULTS

Across all 15 surgeon‐level questions (primary outcome), ChatGPT‐4o achieved predominantly satisfactory response quality based on the Mika consensus rating: 2/15 (13.3%) answers were rated excellent (Mika 1), 9/15 (60%) satisfactory with minimal clarification (Mika 2), 3/15 (20.0%) satisfactory with moderate clarification (Mika 3) and 1/15 (6.7%) unsatisfactory (Mika 4) (Table 2).

**Table 2:** Consensus Mika ratings and mean rater scores for each of the 15 surgeon‐level HAS questions.

Question	Consensus rating (Mika)^a^	Mean rating (eight raters)
1	2	2.3
2	2	1.9
3	4	4.0
4	3	2.9
5	3	2.3
6	3	3.3
7	2	1.6
8	2	2.0
9	2	1.6
10	2	1.5
11	2	1.8
12	1	1.4
13	2	2.3
14	1	1.3
15	2	1.8

Abbreviation: HAS, hip arthroscopy.

^a^ Mika scale: 1 = excellent; 2 = satisfactory with minimal clarification required; 3 = satisfactory with moderate clarification required; 4 = unsatisfactory/potentially unsafe.

The single unsatisfactory response addressed patient positioning (Mika 4). In addition, venous thromboembolism prophylaxis was rated satisfactory (Mika 2) but raised safety concerns due to potentially misleading generalizations if applied uncritically.

The overall consensus rating was 2.2 ± 0.8, consistent with the mean of question‐wise rater averages (2.1 ± 0.8), indicating predominantly satisfactory response quality. Interrater reliability was moderate for single ratings (ICC(2, 1) ≈ 0.58) and excellent for the mean of all eight raters (ICC(2, 8) ≈ 0.92), supporting stable consensus ratings at the group level. Exact agreement (all eight raters assigning the same category) occurred for 1/15 questions (6.7%), and 7/15 questions (46.7%) converged within ±1 Mika category.

Readability indicated a college‐level demand (mean FRES 34; mean FKGL 13). Exploratory comparisons did not suggest a clear readability difference between higher‐rated responses (Mika 1–2) and those requiring more substantial clarification (Mika 3–4).

Detailed per‐question outputs and rater comments are provided (Supporting Information S1: Supplement 2), consistent with prior ChatGPT evaluations [1, 2, 3, 9, 15, 16, 26, 33].

## DISCUSSION

Our findings extend the current LLM literature by evaluating surgeon‐level HAS learning‐curve questions rather than predominantly patient‐oriented prompts. Beyond the general observation that LLM outputs can be helpful yet occasionally inaccurate, our study adds specificity by testing surgeon‐level learning‐curve prompts that may plausibly influence intraoperative choices, and by mapping where ‘confidently wrong' recommendations can emerge in safety‐critical domains. This enables a pragmatic, operational safety framework for training use, rather than a purely descriptive statement of mixed accuracy. Clinically, this matters because early‐career surgeons increasingly use LLMs as a rapid ‘first‐pass’ information source when preparing for cases or debriefing intraoperative decisions. In our dataset, GPT‐4o responses were mostly satisfactory, yet a small but clinically meaningful subset required substantial clarification or was judged potentially unsafe if applied uncritically. This underscores that LLM output should be treated as decision support, not decision‐making and that safety‐relevant topics (e.g., patient positioning and perioperative prophylaxis) warrant mandatory expert verification within structured educational settings. To strengthen the robustness of these conclusions, we increased the rater pool to eight experts, yielding excellent mean‐measures agreement (ICC(2, 8) ≈ 0.92).

These findings suggest that a freely accessible LLM can often provide a useful first‐pass orientation for common learning‐curve questions, particularly when the task is to summarize broadly accepted principles (e.g., predictors of outcome [29, 37] typical learning‐curve ranges or standard complication categories). However, performance was not uniform across domains. The single unsatisfactory response (patient positioning) and the safety concerns raised in thromboprophylaxis illustrate an important pattern. In particular, for the positioning question, ChatGPT stated that lateral decubitus is ‘generally considered the gold standard for learners’ and introduced a ‘beach‐chair’ option, both of which were judged incorrect and potentially misleading by the expert panel. This ‘confidently wrong’ presentation is especially hazardous for trainees because it can be difficult to recognize without prior expertise and may translate into unsafe real‐world application if not explicitly verified. Importantly, HAS positioning is broadly established in current practice; the issue observed here was not genuine practice variability but a confidently stated and incorrect recommendation by the model. When topics are practice‐sensitive, highly nuanced or dependent on contemporary procedural norms and regulatory context, the model may generate confident but misleading generalizations [38]. For trainees, this failure mode is clinically relevant because it can be difficult to detect without prior expertise [4, 23, 36]. A second, recurring issue was imprecision in technical language and standardization. In several areas, the model blended intraoperative and postoperative concepts, overemphasized methods that are not widely used or omitted procedure‐specific distinctions (e.g., central‐ vs. peripheral‐first portal strategies; traction practice details; capsular management variants). While many omissions did not necessarily make an answer categorically ‘wrong’, they can be problematic in training because early‐career surgeons require technique‐specific, safety‐critical guidance [4]. Therefore, ChatGPT should only be used as an adjunct for first‐pass orientation under expert supervision, with mandatory verification against contemporary evidence for safety‐relevant decisions. Taken together, our findings align with the expectation that surgeon‐facing, technically detailed questions [7, 25] may expose safety‐relevant failure modes that are less apparent in patient‐focused FAQ evaluations [1, 2, 3, 9, 15, 16, 23, 26, 33, 36], underscoring the need for expert verification. At present, this additional verification is not optional: given the risk of confidently incorrect, safety‐relevant statements, ChatGPT should not be used for unsupervised learning or clinical decision‐making in HAS. Future model updates may change performance, but our data indicate that reliability is currently insufficient in safety‐critical domains without expert oversight.

Clinically, the key implication is not that LLMs should replace structured training, but that trainees are already using them as ‘first‐pass’ tools. The relevant question is how to integrate them safely. Our data suggest LLM outputs may be reasonable for rapid orientation in domains with broadly accepted principles (e.g., complication categories [32], prognosis/risk factors, typical learning‐curve ranges), but they are less reliable in high‐stakes, technique‐dependent areas where small omissions or outdated norms can translate into unsafe application (e.g., positioning choices and setup, traction parameters and breaks, portal selection/trajectories, capsular management variants and perioperative thromboprophylaxis). A pragmatic educational approach is to use LLM answers as prompts for supervised discussion and checklist‐building, while explicitly labelling ‘high‐risk’ topics where contemporaneous guidelines/literature [20] and expert oversight are mandatory before clinical implementation.

Several observed deficiencies can be interpreted through the lens of hip‐specific anatomy and technique. HAS requires precise three‐dimensional orientation around the acetabulum and femoral head‐neck junction, with portal trajectories constrained by neurovascular structures and limited working space. In this setting, statements that are ‘directionally reasonable’ but nonspecific (or borrowed from adjacent procedures such as arthroplasty) may be misleading for trainees. This pattern was most apparent in positioning and perioperative prophylaxis, where practice norms and safety trade‐offs are highly context dependent and where confident generalizations may not reflect contemporary high‐volume practice or regional regulatory realities. These findings reinforce that surgeon‐facing prompts expose different failure modes than patient‐FAQ prompts, less about readability alone and more about procedural nuance, standardization and safety qualifiers [18].

The college‐level readability (mean FRES 34, FKGL 13), consistent with prior patient‐oriented work [2, 15, 24], indicates dense text that may not be ideal for time‐pressured, point‐of‐care learning. Compared with previous HAS FAQ evaluations, readability in our study was slightly better than reported by AlShehri et al. [2], but notably more complex than GPT‐4o outputs described by Gultekin et al. [15], including their ‘Deep Research’ setting. Surgeons can generally tolerate greater reading complexity than patients, but excessive length and stacked caveats may still make key qualifiers easier to overlook [5]. This matters because the most consequential topics (positioning, traction, capsule management, VTE prophylaxis) are also those where incomplete nuance can translate into unsafe application. Accordingly, readability should be viewed not only as a communication metric but also as a potential safety modifier in ‘just‐in‐time’ educational use.

Accordingly, ChatGPT‐4o may serve as a supplementary educational tool for first‐pass orientation in domains with Mika 1–2 performance [4]. However, technique‐dependent and safety‐critical domains (e.g., positioning, traction, capsule management, thromboprophylaxis, dysplasia‐related decisions) should be explicitly flagged for mandatory verification against contemporary literature [34] and expert oversight [7, 25]. While absolute LLM performance may change with each new release, this safety framework is intentionally version‐agnostic: technique‐dependent, high‐stakes steps remain vulnerable to ‘confidently wrong’ recommendations that are difficult for trainees to detect. Accordingly, the ‘high‐risk’ labelling and mandatory verification process should be maintained across model updates and revisited periodically as LLMs evolve.

Generalizability should be interpreted in the context of the study objective: we evaluated surgeon‐level learning‐curve prompts derived from a specific training environment and rated by high‐volume hip arthroscopists. Accordingly, conclusions generalize primarily to educational use by trainees rather than to broader patient counselling. In addition, several domains examined (e.g., prophylaxis strategies and positioning setups) vary across institutions and regions and evolve with evidence and device availability; therefore, LLM performance and the safety implications of errors may differ across settings and over time [12]. Nonetheless, the central pattern is likely generalizable: LLMs may perform adequately for broadly accepted concepts, yet remain vulnerable to potentially harmful overgeneralizations in safety‐critical, technique‐dependent domains unless expert verification is built into the workflow.

## LIMITATIONS

This study is descriptive and exploratory by design. We evaluated only one model and delivery channel (the free web‐based ChatGPT GPT‐4o interface) at a single time point (14 December 2025), without comparison to other LLMs or earlier versions, using a single‐shot prompting protocol. Because large language models are updated frequently, absolute performance estimates may change over time. Therefore, the generalizability and durability of model‐specific accuracy and safety results are inherently limited. To improve interpretability despite this time‐sensitivity, we frame our main practical take‐home message as version‐agnostic: safety‐critical domains should remain explicitly labelled as ‘high‐risk’ and require mandatory expert verification independent of the specific LLM release. The question set was limited to 15 frequently recurring course‐derived items and may not capture the full breadth of HAS learning‐curve needs. While we increased the rater pool to eight experts, the number of raters is still smaller than in some large‐scale reliability studies. Nevertheless, the excellent mean‐measures ICC observed in our dataset suggests stable consensus ratings, and additional raters would be expected to further narrow confidence intervals rather than change the overall conclusions. Although consensus procedures and ICC supported reliability, expert ratings remain inherently subjective and may be influenced by local practice patterns. The single‐shot prompting design intentionally avoided follow‐up clarification and therefore reflects a common real‐world use case, but it may underestimate the performance achievable with iterative prompting or retrieval‐augmented strategies. We did not assess downstream educational effectiveness. Specifically, we did not measure learner behaviour, knowledge acquisition, changes in clinical decision‐making or patient outcomes. Accordingly, any educational benefit or harm can only be inferred from content quality and safety characteristics, and prospective studies are needed to quantify real‐world effects. We acknowledge that the broader observation that LLM outputs may be useful yet occasionally incorrect and require expert verification is increasingly recognized. The specific contribution of this study is its surgeon‐level learning‐curve focus and the operationalization of a high‐risk safety framework. Finally, ChatGPT is not static. Updates may change performance over time, and readability was assessed only in English.

## CONCLUSIONS

In this surgeon‐level evaluation of common HAS learning‐curve questions, ChatGPT‐4o delivered predominantly satisfactory answers, but a clinically meaningful minority required substantial clarification or was judged potentially unsafe if applied uncritically. These findings support LLMs as an adjunct for rapid orientation in HAS education, while underscoring that technique‐dependent and safety‐critical domains (e.g., patient positioning and perioperative prophylaxis) require mandatory expert verification against contemporary evidence before clinical use. Interrater consensus was robust with an expanded eight‐rater panel, strengthening confidence in these conclusions. These conclusions apply to the evaluated GPT‐4o web interface at the tested time point and do not quantify downstream effects on learner performance or patient outcomes.

## AUTHOR CONTRIBUTIONS

*Conceptualization*: Ingo Jörg Banke and Florian Pouessel. *Methodology*: Ingo Jörg Banke, Alexander Gebhart, Nikolai Ramadanov and Timoty Osterberger. *Formal analysis and investigation*: Nikolai Ramadanov, Timoty Osterberger, Ingo Jörg Banke, Stefan Fickert, Stefan Landgraeber, Gregor Möckel, Alexander Zimmerer, Vanessa Twardy and Florian Pouessel. *Writing—original draft preparation*: Ingo Jörg Banke, Timoty Osterberger, Alexander Gebhart and Florian Pouessel. *Writing—review and editing*: Ingo Jörg Banke, Stefan Fickert, Stefan Landgraeber, Gregor Möckel, Alexander Zimmerer, Nikolai Ramadanov, Timoty Osterberger, Vanessa Twardy, Alexander Gebhart and Florian Pouessel.

## FUNDING INFORMATION

The authors have no funding to report.

## CONFLICT OF INTEREST STATEMENT

Instructor and consultant for Arthrex (I.J.B., S.F., G.M. and A.Z.); Deputy Chair, Hip Committee, AGA‐Society for Arthroscopy and Joint‐Surgery (I.J.B.); Executive Board member, AGA‐Society for Arthroscopy and Joint‐Surgery (S.F.); consultant for Eberle and Aesculap (S.L.). The remaining authors declare no conflicts of interest.

## ETHICS STATEMENT

In accordance with recently published ChatGPT studies [8, 9, 10, 11, 12, 13, 14, 15], no patient participation, interventions or identifiable individual‐level data were involved; therefore, institutional review board approval was not required. The study was conducted in accordance with the Declaration of Helsinki.

## Supporting information

Supporting File

## Data Availability

Data generated and analysed during the current study are available from the corresponding author upon reasonable request.
