# Supplementary material for: ChatGPT delivers mostly satisfactory but occasionally inaccurate and potentially unsafe answers to hip arthroscopy questions during the surgeon's learning curve
Source: J Exp Orthop. 2026 May 25;13(2):e70770. doi: 10.1002/jeo2.70770 (PMC13239738; doi:10.1002/jeo2.70770)
Supplement: Supplementary file 1 — Supporting File [file JEO2-13-e70770-s001.docx]

**Supplement 1.** ChatGPT Response Rating System by Mika et al. [23]

| **Response Accuracy Score** | **Response Accuracy Description** |
| --- | --- |
| **1** | **Excellent response not requiring clarification** |
| **2** | **Satisfactory requiring minimal clarification** |
| **3** | **Satisfactory requiring moderate clarification** |
| **4** | **Unsatisfactory requiring substantial clarification** |

**Supplement 2.** Questions Prompted to ChatGPT, ChatGPT Responses, Response Accuracy Scores Assigned by Expert Reviewers, and Brief Reviewer Comments

| **Question 1** | **Which types of hip arthroscopy cases are most appropriate**  **for surgeons early in the learning curve?** |
| --- | --- |

**ChatGPT response:** Selecting appropriate early cases is crucial for safely developing hip arthroscopy skills given the steep learning curve and limited intra-articular working space. Early cases should be technically straightforward, with clear indications, good joint access, and minimal anatomic distortion; complex pathology and revisions should be deferred until access, visualization, and instrumentation are reliable. Suitable initial cases include isolated cam-type femoroacetabular impingement with minimal acetabular overcoverage, which typically allows easier peripheral-compartment access for cam resection and good visualization of the head–neck junction, ideally in young, thin, non-dysplastic patients with limited cartilage damage. Isolated labral tears with minimal chondral injury are also appropriate, enabling practice of portal placement, diagnostic arthroscopy, and basic labral repair or debridement once access is achieved; ideal patients lack significant pincer morphology, dysplasia, or advanced osteoarthritis (Tönnis 0–1). Diagnostic arthroscopy or loose body removal are favorable early cases as short, focused procedures that build familiarity with traction setup, joint anatomy, and scope handling with relatively low iatrogenic risk.

Cases to avoid early include complex combined cam/pincer FAI with deep overcoverage, dysplasia or borderline dysplasia (instability risk), revision arthroscopy, extensive chondral damage or labral reconstruction, severe obesity or limited distraction, and cases after periacetabular osteotomy or prior hip surgery. Additional recommendations include cadaveric/simulation training before live surgery, early mentorship or proctoring by an experienced hip arthroscopist, and more frequent fluoroscopic use to support safe portal placement and minimize iatrogenic injury.

**Response Grade:** 2, satisfactory requiring minimal clarification.

**Analysis:** Experts considered the progression from simple to complex cases meaningful and noted that activity level and body weight were mentioned. However, the response underemphasized cam morphology/femoroacetabular impingement syndrome (FAIS) and did not clearly address osteoarthritis as a key limiting factor [29]. Per the Warwick Agreement, terminology should refer to FAIS and bony morphology (not “lesions”), distinguishing cam, pincer, and combined types [14]. Cam location (anterolateral vs lateral/posterolateral), critical for beginner accessibility, was omitted. Overall, the answer was adequate but only moderately structured and incompletely justified.

| **Question 2** | **What preoperative imaging is recommended before hip arthroscopy?** |
| --- | --- |

**ChatGPT response:** Preoperative imaging is essential for planning hip arthroscopy, as it helps characterize joint status and pathology that may affect indications and outcomes. Plain radiographs are the first-line screening tool to assess alignment, degenerative changes, dysplasia, and bony morphology relevant to FAI. Common views include an AP pelvis, a frog-leg lateral, and a cross-table lateral to evaluate the femoral head–neck junction and acetabulum.

MRI is recommended to evaluate soft tissues and intra-articular pathology, including labral tears, cartilage damage, ligamentous injury, synovitis, and related abnormalities. MRI may be performed with or without contrast; an MR arthrogram can improve visualization of the labrum and cartilage in selected cases.

Computed tomography (CT) provides high-detail assessment of bony anatomy and can be useful for complex cases and preoperative planning (e.g., femoral/acetabular osteoplasty) where precise 3D characterization of morphology is required. It may also be used postoperatively to assess bony correction when needed.

Ultrasound is not routinely used for preoperative structural assessment, but it can support image-guided diagnostic intra-articular injections to confirm an intra-articular pain source when diagnostic uncertainty exists.

Overall, typical preoperative imaging includes radiographs for bony morphology/alignment, MRI/MRA for labrum and cartilage, CT when detailed bony planning is required, and ultrasound mainly to facilitate diagnostic injections, tailored to the suspected pathology.

**Response Grade:** 2, satisfactory requiring minimal clarification

**Analysis:** Overall, experts found the question and response broadly useful, but felt limitations of modalities were underappreciated. Magnetic resonance imaging (MRI)’s role for labral and chondral assessment was not emphasized enough, while CT was somewhat overrated and radiation exposure was not mentioned. Key technical elements were missing, including a correctly centered AP pelvis (on the symphysis), the value of the Dunn 45° view for cam detection, and the distinction between 1.5T MRA vs high-resolution 3T MRI as recommended in the Lisbon Agreement [20]. The response also omitted rotational MRI/CT considerations and contained errors, including the implication that labral tears are detectable on plain radiographs, plus only marginally relevant remarks on ultrasound-guided injections.

| **Question 3** | **What patient positioning is recommended for surgeons**  **early in the hip arthroscopy learning curve?** |
| --- | --- |

**ChatGPT response:** Early in the hip arthroscopy learning curve, patient positioning is critical for safety, comfort, and optimal joint access. The two most common positions are lateral decubitus and supine; however, the lateral decubitus position is most frequently used and is generally recommended for beginners. In lateral decubitus, the patient lies on the side with the hip flexed (often ~90°) and the leg slightly abducted, which can facilitate access to anterior and posterior aspects of the hip for procedures such as labral repair, femoroacetabular impingement correction, and cartilage debridement. Reported advantages of lateral decubitus especially for learners include excellent anatomical access with minimal risk to vital structures, improved visualization, easier identification of landmarks and instrument guidance, effective traction application to create joint space, and significantly lower nerve-injury risk, particularly to the femoral or sciatic nerves, when performed correctly.

The supine position is an alternative with the patient on the back and the affected leg slightly abducted and externally rotated, using traction to open the joint space before portal placement. For learners, the supine position offers a simple setup and provides direct anterior access to the hip joint, which can be beneficial for labral repair or impingement resection, with traction often easier to manage; however, it can limit access to posterior structures and may be less suitable for complex procedures requiring circumferential visualization.

A third option, the modified beach chair position, places the patient upright seated with hips flexed and legs supported; it may reduce nerve-injury risk compared to other positions and offers a favorable angle for femoroacetabular impingement work or anterior labral pathology.

Regardless of position, early learners must prioritize patient safety and comfort (padding and secure fixation to prevent pressure sores, nerve injury, and respiratory compromise), apply traction correctly to create joint space and maintain visualization, and learn to titrate the required traction. Portal placement is position-dependent (lateral decubitus: anterior and anterolateral portals; supine: often an anterior portal only), and beginners must know standard portal locations and adapt them to the surgical goal.

Overall, although both lateral and supine positions are used, lateral decubitus is generally considered the gold standard for learners because it improves anterior/posterior access, visibility, and maneuverability; supine and beach chair may suit selected cases (e.g., anterior labral tears or impingement resection) but can be more challenging early on. With increasing experience, surgeons can expand to alternative positioning based on patient and procedure needs.

**Response Grade:** 4, **unsatisfactory requiring substantial clarification.**

**Analysis:** All experts agreed the response incorrectly favors lateral decubitus and contains false or contradictory statements. In current practice, the supine position is the worldwide standard for hip arthroscopy, and any general superiority of lateral positioning could not be substantiated [8]. The stated pros/cons of lateral versus supine positioning were poorly supported, key elements such as postless techniques were omitted, and nerve-injury risk was discussed in a confusing and partly incorrect manner. The response also inappropriately endorses a beach-chair–type position, which is not used in contemporary hip arthroscopy: the semi-seated posture limits hip extension and makes central-compartment access difficult, while increasing genital pressure against the perineal post with presumed higher pudendal nerve risk. Overall, the recommendation was considered misleading and unacceptable for surgeons in the learning curve.

| **Question 4** | **What standard portal placement is recommended for hip arthroscopy?** |
| --- | --- |

**ChatGPT response:** In hip arthroscopy, standard portal placement is essential for visualization and instrument access. The anterolateral portal is typically placed about 2–3 cm distal and 2–3 cm anterior to the anterior superior iliac spine and is commonly used for the camera, providing a relatively neutral view of the joint. The anteromedial portal is usually positioned just medial to the anterior inferior iliac spine and roughly 2–3 cm distal to the inguinal ligament; it is frequently used for instruments (e.g., shavers, probes) and facilitates access to central and medial joint regions. A posterolateral portal may also be used, typically just posterior to the greater trochanter and slightly inferior to the posterior acetabular rim, to access posterior structures for procedures such as labral repair or iliopsoas tendon release, although it is not required in all cases. The mid-anterior portal is an additional variation but is less common than the primary anterolateral and anteromedial portals.

Safe portal placement relies on accurate anatomic landmarks to avoid injury to structures such as the femoral nerve, artery, and vein. Patient positioning (supine with slight hip flexion/abduction or modified lateral) influences portal orientation and choice. Fluoroscopy may be used selectively to confirm portal placement, particularly when working near sensitive structures. Together, these portals provide access for diagnostic and therapeutic interventions, including treatment of labral tears, femoroacetabular impingement, and loose-body removal.

**Response Grade:** 3, satisfactory requiring moderate clarification.

**Analysis:** Experts agreed, although some basic aspects of portal placement for a central-compartment–first technique were mentioned, the portal description was confusing, incomplete, and partly incorrect. It failed to distinguish central-first from peripheral-first approaches and omitted key peripheral-first portals (proximal anterior/proximal anterolateral) as well as the DALA portal, which is useful for labral and cartilage work. Several localizations (anterolateral/anteromedial) were inaccurate, and relevant safety issues, such as sciatic nerve risk with a posterolateral portal in internal rotation [27], were not addressed. Overall, the answer was too general and insufficiently differentiated for surgeons in the learning curve. Overall, the answer was judged too general and undifferentiated for surgeons in the learning curve.

| **Question 5** | **How long is traction safe during hip arthroscopy?** |
| --- | --- |

**ChatGPT response:** In hip arthroscopy, traction is used to distract the joint and create working space for procedures such as labral repair, cartilage treatment, or impingement correction. Traction duration depends on case complexity and patient anatomy, but is typically maintained for 20-45 minutes, which is generally considered safe. Factors influencing duration include the surgical approach, underlying pathology, and patient-specific considerations (e.g., anatomic variation or comorbidities), and more complex procedures may require longer traction times. Safety concerns increase when traction is prolonged beyond ~45 minutes, as extended traction may raise the risk of complications such as nerve injury (particularly femoral nerve), muscle injury, or circulatory problems. These risks can be mitigated by continuous intraoperative monitoring, and traction may be applied intermittently or adjusted as needed. Overall, traction is considered safe for the time required by the procedure, typically under 45 minutes, when appropriately monitored, and specific concerns should be discussed with the operating surgeon.

**Response Grade:** 3, satisfactory requiring moderate clarification.

**Analysis:** Experts found the 20-45 min estimate broadly acceptable but potentially misleading, as no study-defined “optimal” traction limit exists and expert opinion often allows up to ~60 min, especially for novices [35]. Key omissions included traction-release breaks (30-45 min), using minimal traction force, meticulous perineal-post/soft-tissue padding, postless options, and documenting traction start/pauses/end. The nerve-injury section was also off: femoral traction injury is unlikely, whereas pressure-related pudendal nerve injury is the main concern. Overall, the response was partially correct but too incomplete and imprecise for trainees without clarification.

| **Question 6** | **For femoroacetabular impingement syndrome, which capsular**  **management is generally recommended in hip arthroscopy?** |
| --- | --- |

**ChatGPT response:** In femoroacetabular impingement syndrome (FAI), capsular management during hip arthroscopy is important for optimizing outcomes and minimizing postoperative instability. In most cases, capsular repair is generally recommended, reapproximating the capsule to restore its anatomic integrity, because this helps maintain stability, reduces the risk of postoperative instability or dislocation, and may support long-term outcomes by limiting instability and loss of motion.

When there is marked laxity or a large capsular defect, capsular plication may be performed to tighten the capsule and reduce laxity. A capsulotomy is often required to access the joint for labral repair or femoral head–neck osteoplasty; after completing the procedure, the capsule is typically sutured closed to restore structural integrity. An anterior capsulotomy is commonly used for FAI work, and in cases with concern for instability, particular emphasis is placed on meticulous closure. Capsular release is less frequent but may be considered when significant capsular contracture or stiffness is present, such as after prior FAI surgery, to improve range of motion.

Technique selection depends on factors such as preoperative instability, degree of stiffness, and surgeon preference, while avoiding excessive capsular resection or inadequate repair that could increase instability. Overall, capsular repair is generally favored in hip arthroscopy for FAI, with plication or selective release used in individualized situations.

**Response Grade:** 3, satisfactory requiring moderate clarification.

**Analysis:** Experts appreciated the “safety first” tone in a controversial field, but found the response insufficiently differentiated, confusing, and partly conflated with total hip arthroplasty concepts. It implied capsular repair is routinely essential, whereas many hip arthroscopy indications do not require extensive capsulotomy or release and capsule-preserving techniques should be prioritized, especially for beginners. Key strategies (puncture/dilation vs periportal vs T-shaped capsulotomy) were not distinguished; evidence suggesting preferential closure of T-capsulotomy, frequent non-closure of periportal capsulotomy, and mandatory repair in borderline dysplasia was not reflected [30]. Overall, the text was considered inconsistent, poorly structured, and not aligned with contemporary high-volume practice.

| **Question 7** | **In hip arthroscopy, when is labral repair preferred over debridement?** |
| --- | --- |

**ChatGPT response:** In hip arthroscopy, the choice between labral repair and debridement depends on tear characteristics, tissue quality, and patient goals. Repair is generally preferred when the labrum is sufficiently healthy and structurally intact, particularly for stable peripheral tears near the acetabular attachment and in the anterior/superior weight-bearing regions where reattachment is feasible. Preserving the labrum helps maintain hip stability, cushioning, and proprioception and is therefore favored in young, athletically active patients to support long-term function and joint preservation, potentially reducing osteoarthritis risk.

In femoroacetabular impingement or hip dysplasia, repair is preferred when the labrum remains relatively intact and its anatomic function can be restored, ideally combined with correction of the underlying bony abnormality to limit degeneration. Repair is also more suitable for less complex tear patterns with minimal degeneration and limited cartilage damage (e.g., acute or traumatic tears) that allow secure fixation. Conversely, debridement is more often considered when tissue is frayed, degenerative, or irreparable, such as in advanced osteoarthritis, or when tears are extensive or complex and cannot be reliably repaired. While Research suggests that repair provides better long-term outcomes in pain, function, and joint preservation, debridement may offer short-term symptom relief but does not restore labral function and may increase osteoarthritis risk in younger or more active patients. Overall, repair is preferred when reattachment is feasible and long-term preservation is desired, whereas debridement is reserved for non-repairable tissue.

**Response Grade:** 2, satisfactory requiring minimal clarification.

**Analysis:** Experts agreed with the strong emphasis on labral preservation and felt the main principles were covered, particularly if “dysplasia” is interpreted as borderline dysplasia, but noted repetition and occasional imprecision [18]. The response did not clearly state that isolated arthroscopy for labral pathology in true dysplasia is insufficient and requires bony correction, nor that current data show no added benefit of combining pelvic reorientation osteotomy with arthroscopy for labral pathology. It also lacked guidance for ossified labra. Overall, the answer was broadly acceptable but required targeted refinement.

| **Question 8** | **During hip arthroscopy, how can adequacy of cam resection be assessed?** |
| --- | --- |

**ChatGPT response:** Adequate cam resection in hip arthroscopy is critical to restore joint mechanics and prevent ongoing impingement. Intraoperatively, assessment relies on several complementary methods. Direct visualization of the femoral head-neck junction helps confirm that the cam prominence has been removed while preserving femoral neck integrity; the goal is a smooth, round head with a continuous head-neck transition without residual bumps.

Intraoperative imaging, most commonly fluoroscopy, provides real-time confirmation of resection adequacy. Standard AP and lateral views are used to evaluate the head–neck contour and confirm correction without residual prominence; CT may be used in select settings intra- or postoperatively when available.

Dynamic assessment also contributes. After resection, the surgeon can perform range-of-motion and impingement testing (e.g., FADIR) and passively move the hip to detect residual bony conflict or abnormal contact. Tactile feedback (e.g., probing/palpation) can help identify step-offs or remaining prominence requiring refinement. In some cases, guides/templates or computer-assisted navigation may support estimation and precision of bone removal.

Key principles include avoiding over-resection, which can weaken the femoral neck and increase fracture risk, while ensuring sufficient correction and maintaining femoral head sphericity. Maintaining the sphericity of the femoral head is essential for normal hip biomechanics and reducing the risk of further joint degeneration. Overall, adequacy is judged through a combination of visualization, imaging, tactile assessment, and dynamic testing.

**Response Grade:** 2, satisfactory requiring minimal clarification.

**Analysis:** Experts found the response largely correct and sufficiently detailed but noted inaccuracies and overemphasis on uncommon methods. It partly conflated intraoperative assessment with postoperative testing (FADIR cannot be performed under general anesthesia), referenced non-routine tools (e.g., a “soft probe”), and portrayed navigation as standard when it is not. The answer also did not align clearly with the Warwick Agreement definition of cam morphology or with commonly used standardized imaging protocols (multiple AP and lateral views at varying flexion/rotation) [14]. Overall, it was generally useful but required correction of these technical points and a clearer focus on widely available techniques.

| **Question 9** | **During hip arthroscopy, how can adequacy of pincer resection be assessed?** |
| --- | --- |

**ChatGPT response:** Assessing adequacy of pincer resection during hip arthroscopy for femoroacetabular impingement is essential to ensure acetabular overcoverage is corrected without causing instability. A primary method is direct visualization of the acetabular rim after rim trimming, aiming for a smooth, regular contour while avoiding over-resection (instability) and under-resection (residual impingement). Intraoperative fluoroscopy is commonly used to confirm adequacy by comparing pre- and post-resection images (e.g., AP pelvis, 45° Dunn, false-profile) against preoperative planning goals such as improved head coverage. Surgeons may assess radiographic features such as reduction of the crossover sign, correction of cranial retroversion, restoration of a more normal acetabular contour, and preservation of sufficient coverage; calibrated templates may assist with estimating rim trimming.

Additional checks include visual or tactile assessment for residual bony overhang or sharp edges that could still impinge during motion. Some centers use navigation or 3D imaging to guide rim trimming and provide real-time anatomic feedback. Adequacy also involves labral preservation: the labrum should remain intact when possible, with labral repair performed if needed. Range-of-motion/impingement testing during passive movement may help identify persistent impingement suggesting insufficient resection. Overall, adequacy is judged through visualization, fluoroscopy, assessment for residual overhang, and, in selected settings, navigation and dynamic testing, balancing impingement relief with stability and labral preservation.

**Response Grade:** 2, satisfactory requiring minimal clarification.

**Analysis:** Experts found the response generally good, detailed, and summarizing key points well. Minor refinements were missing: fluoroscopy should be specified as a C-arm PA view centered on the hip, with acknowledgment of its limitations, and “pincer morphology” is preferable to “pincer deformity” [14]. The answer also referenced postoperative-only measures (e.g., pain on testing), which are not strictly intraoperative. Overall, it was considered largely complete and clinically useful for surgeons in the learning curve

| **Question 10** | **What intraoperative complications are most common during hip arthroscopy?** |
| --- | --- |

**ChatGPT response:** Hip arthroscopy is generally safe, but several intraoperative complications can occur related to positioning, traction, portals, and instrumentation. Nerve injuries are among the most frequently discussed, including irritation of the lateral femoral cutaneous nerve from traction, instrument contact, or compression; obturator nerve injury is rare; and sciatic nerve injury is very uncommon but may occur with excessive traction or positioning. Vascular injuries are less common but potentially serious, including damage to the femoral artery or vein from improper instrument placement or traction, with possible bleeding or compromised limb perfusion. Although primarily peri- and postoperative, deep venous thrombosis risk is also noted in the context of prolonged positioning/immobilization. Infection remains a general surgical risk, including superficial and deep infection after hip arthroscopy.

Iatrogenic labral or cartilage injury can occur during portal placement, diagnostic exploration, or interventions (debridement/repair), affecting the labrum or acetabular/femoral head cartilage. Soft-tissue and traction-related injuries (strain to hip/groin/thigh) may prolong recovery and can include muscle, tendon, or ligament injury. Fractures of the acetabulum or femur are rare but serious, typically from excessive force or instrument-related trauma. Other reported complications include hematoma related to traction, rare hip dislocation with improper force/positioning, and uncommon chondrolysis potentially related to irrigation issues or thermal injury. Anesthetic complications (e.g., hypotension, allergic reactions) may also occur, particularly with regional techniques.

Prevention centers on meticulous positioning and padding, careful traction management and monitoring, accurate portal/instrument placement, and controlled instrumentation; fluoroscopy may assist with safe access and early recognition of problems. By adhering to best practices and maintaining awareness of potential complications, the risks associated with hip arthroscopy can be minimized.

**Response Grade:** 1, **excellent response not requiring clarification.**

**Analysis:** Experts found the complication list detailed, well structured, and surprisingly accurate, though it included some very rare events. Missing or underemphasized risks included under-/overresection [32], bleeding compromising visibility, femoral head necrosis after vascular injury, dislocation after excessive capsular surgery, and rare but serious complications such as traction-related compartment syndrome, tendon injury (e.g., iliopsoas), equipment failure, and pressure injuries to the scrotum/labia. Overall, despite these gaps, the response was considered comprehensive and highly useful for surgeons in the learning curve, helping to raise awareness of relevant intraoperative risks.

| **Question 11** | **After hip arthroscopy, what postoperative rehabilitation protocol**  **is recommended?** |
| --- | --- |

**ChatGPT response:** Postoperative rehabilitation after hip arthroscopy is essential for recovery, joint protection, and return to function, but protocols vary by indication and procedures performed (e.g., labral repair, FAI correction, microfracture). Core goals are to protect repairs, restore range of motion (ROM) progressively, rebuild neuromuscular control, strength, and stability, normalize gait, and enable gradual return to sport.

0-2 weeks (acute phase): focus on pain/inflammation control, protection of repairs, gentle ROM, and early muscle activation. Weight bearing is typically partial with crutches (often ~20-30% BW); after microfracture, toe-touch weight bearing is common. ROM precautions often include avoiding flexion >90° and limiting extension/external rotation depending on surgeon preference; a brace may be used. Active hip flexion is often avoided early. Typical exercises include passive ROM/circumduction, isometrics (gluteal/quadriceps sets), core activation, and stationary cycling without resistance if tolerated.

2-6 weeks (early rehab): gradual ROM restoration, initiation of gentle strengthening, and progression toward full weight bearing as tolerated; crutches are commonly discontinued by 4–6 weeks once gait normalizes. Closed-chain exercises (bridges, mini-squats, step-ups), hip/core stability, and optional pool therapy may be added.

6-12 weeks (strengthening): aim for near-full/full ROM, improved strength/endurance, and normalized movement mechanics. Progress resistance training (e.g., leg press, hip ab/adduction, lunges), single-leg balance, and cardiovascular conditioning (bike/elliptical), with gentle proprioception/agility drills.

3-6 months (return to activity): progress dynamic stability and sport-specific work. Running may begin around 3-4 months if pain-free with ~90% strength vs the contralateral side; plyometrics often start 4-5 months, and return to sport is commonly 4-6 months, depending on intraoperative findings and recovery.

General guidance is to avoid high-impact activity until later stages (often 4-6 months) and individualize progression to procedure and patient factors. Procedure-specific modifications include longer protected weight bearing after microfracture (6-8 weeks toe-touch with gradual progression/CPM) and typical partial weight bearing for 2-4 weeks after labral repair/FAI osteoplasty or capsular plication, with early ROM restrictions (e.g., limiting external rotation/extension). Monitoring for red flags (increasing pain/swelling, instability/limp, or infection signs) is recommended.

**Response Grade:** 2, satisfactory requiring minimal clarification.

**Analysis:** Experts found the response extensive, broadly evidence-based, and aligned with current recommendations, with a clear phase-based structure [34]. The main drawback was a “one-size-fits-all” protocol that insufficiently differentiated by indication and procedure: rehab after minor labral/pincer pathology or synovial chondromatosis can be much more liberal than after advanced cartilage surgery. It also blurred microfracture versus cartilage transplantation/regenerative techniques, risking inappropriate assumptions about restrictions. Several experts would prefer multiple procedure-specific protocols rather than one generic pathway. Despite this, the key elements were included and coherently presented.

| **Question 12** | **After hip arthroscopy, what prophylaxis is recommended against heterotopic ossification?** |
| --- | --- |

**ChatGPT response:** After hip arthroscopy, heterotopic ossification (HO) is a recognized but relatively uncommon complication involving ectopic bone formation in periarticular soft tissues that can cause pain, stiffness, and reduced mobility. Prophylaxis focuses mainly on pharmacologic measures, with selective use of additional strategies in high-risk patients and attention to perioperative technique.

NSAIDs are most commonly used. Agents such as indomethacin or celecoxib are typically started postoperatively to reduce HO risk; a common approach is initiating indomethacin within 24-48 hours and continuing for 7-14 days, depending on local protocols. NSAIDs act by inhibiting cyclooxygenase-mediated inflammatory pathways implicated in ectopic bone formation.

In selected high-risk patients (e.g., prior HO after hip surgery), low-dose radiation therapy may be considered, usually within 48 hours postoperatively, although its use is controversial due to variable efficacy and concerns about long-term cancer risk.

Early mobilization and physical therapy (gentle ROM) may help limit stiffness and support circulation, but overly aggressive motion should be avoided to prevent excess soft-tissue stress and inflammation. Preventive measures also include minimizing soft-tissue trauma intraoperatively and using controlled bone work/resection to reduce postoperative inflammatory stimuli. Risk factors include prior HO, extensive procedures, family history, instability, and neurologic conditions (e.g., spinal cord injury). Follow-up imaging may be used if symptoms such as increasing pain, stiffness, or loss of ROM develop. Overall, prophylaxis and monitoring should be individualized to patient risk and surgical complexity.

**Response Grade:** 1, **excellent response not requiring clarification.**

**Analysis:** Experts agreed the response was conclusive, well structured, and aligned with current HO prophylaxis recommendations [31]. Minor nuances include that, based on the current evidence, pharmacological prophylaxis should be used routinely after hip arthroscopy, particularly after FAIS osteoplasty, whereas in very limited procedures it may be individualized.

| **Question 13** | **After hip arthroscopy, what venous thromboembolism prophylaxis**  **is recommended?** |
| --- | --- |

**ChatGPT response:** After hip arthroscopy, venous thromboembolism (VTE) prophylaxis aims to reduce the risk of deep vein thrombosis and pulmonary embolism, with the regimen tailored to patient risk factors (e.g., age, obesity, medical history, prior VTE) and bleeding risk. Pharmacologic prophylaxis is commonly used in higher-risk patients. Low-molecular-weight heparin (e.g., enoxaparin) is frequently administered, often for 7-14 days. Direct oral anticoagulants (e.g., rivaroxaban, apixaban) are increasingly used due to oral dosing and no routine monitoring; unfractionated heparin may be considered when LMWH/DOACs are unsuitable.

Mechanical prophylaxis is an important adjunct, including intermittent pneumatic compression in the perioperative/early postoperative period and compression stockings to reduce venous stasis. Early mobilization is strongly encouraged, with early ambulation and simple exercises (ankle pumps, knee flexion/extension) initiated within hours to days per protocol.

Risk stratification using tools such as the Caprini score may guide prophylaxis choice. Some patients may require extended prophylaxis after discharge (e.g., 10–14 days), whereas low-risk patients may be managed with mechanical measures and early mobilization alone. Moderate- to high-risk patients (e.g., obesity, advanced age, prior VTE, prolonged immobility) may benefit from combined pharmacologic and mechanical prophylaxis, individualized to clinical factors and institutional practice.

**Response Grade:** 2, satisfactory requiring minimal clarification.

**Analysis:** Rated satisfactory with minimal clarification, the response raised some safety concerns. It addressed pharmacologic prophylaxis but was overly generic: mandatory LMWH for all patients until full weight-bearing is not hip arthroscopy-specific practice, and DOACs are not approved for arthroscopic procedures in several countries. It largely reflected arthroplasty-style concepts rather than a risk-adapted strategy. Routine IPC devices or compression stockings are not standard [17], and prophylaxis type/duration are individualized and tied to crutch use, typically not exceeding ~4 weeks. Overall, key elements were present, but the lack of procedure-specific nuance could mislead if applied uncritically.

| **Question 14** | **In hip arthroscopy, how many cases are typically required to overcome**  **the main learning curve?** |
| --- | --- |

**ChatGPT response:** In hip arthroscopy, the learning curve is steep due to technical complexity and anatomic constraints. Reported estimates vary by surgeon experience and technique, but many studies suggest roughly 50-100 cases to pass the primary learning curve, with marked gains often after the first ~50 procedures. More advanced proficiency (e.g., complex labral repair, managing FAI or instability) may require ~150-200 cases or more, especially for challenging pathology.

Progress depends on prior minimally invasive experience, case mix/complexity, and the presence of structured mentorship and formal training, which can accelerate skill acquisition. Importantly, proficiency is not defined by case numbers alone, but also by complication management and consistent patient outcomes.

**Response Grade:** 1, **excellent response not requiring clarification.**

**Analysis:** Experts agreed the response was clear, comprehensive, and consistent with recent learning-curve literature. It appropriately highlighted that complex procedures (e.g., labral repair, FAIA, instability) require substantial experience, potentially several hundred cases per published data [22]. A minor omission was the role of annual case volume in maintaining proficiency, but overall the key factors were well covered and well structured.

| **Question 15** | **Which patient factors predict poor outcomes after hip arthroscopy?** |
| --- | --- |

**ChatGPT response:** Predicting poor outcomes after hip arthroscopy, particularly for femoroacetabular impingement or labral pathology, is essential for patient selection and counseling. Outcomes decline with age from ~40 years, and patients >50 years represent a notably unfavorable prognostic group, likely reflecting greater chondral degeneration and reduced healing potential. Some studies associate female sex with worse subjective outcomes, possibly related to greater laxity or smaller alpha angles. Elevated BMI, especially >30, is linked to poorer functional recovery, lower PROMs, and higher revision or conversion rates.

Structural joint status is pivotal. Pre-existing osteoarthritis and joint space narrowing <2 mm are among the strongest predictors of poor outcome and conversion to total hip arthroplasty; Tönnis grade ≥1 is often considered a relative contraindication. Advanced cartilage damage at arthroscopy (Outerbridge III–IV) also predicts worse prognosis. Labral status matters: irreparable tears or debridement (vs repair or reconstruction) correlate with inferior outcomes. Acetabular dysplasia (LCEA <20–25°) may leave residual instability and accelerate osteoarthritis progression, while marked laxity or connective tissue disorders (e.g., Ehlers–Danlos syndrome) predispose to recurrent instability and pain.

Medical and psychosocial factors contribute symptom duration >2 years, depression, anxiety, catastrophizing, workers’ compensation or litigation, and smoking (via impaired soft-tissue healing) are each associated with worse outcomes. Functional and surgical factors also play a role: lower baseline hip function (e.g., reduced preoperative iHOT-33, HOS, or mHHS), inadequate correction of deformity (residual cam/pincer), and revision surgery, which consistently performs worse than primary hip arthroscopy.

**Response Grade:** 2, satisfactory requiring minimal clarification.

**Analysis:** The ChatGPT response was judged overall good and comprehensive, capturing major prognostic factors for hip arthroscopy in a clear, structured manner. The listed risk factors (age, labral status, chondral damage, BMI, sex) broadly reflect the current literature, but several nuances are oversimplified. In particular, the blanket statement that labral debridement leads to worse outcomes is not universally true, as labral resection can yield excellent results in selected constellations (e.g., older female patients with high BMI and pronounced pincer morphology) [37]. Likewise, “joint space <2 mm” is an imprecise standalone criterion and should be replaced by a standardized osteoarthritis classification (e.g., Kellgren–Lawrence). Finally, the question is poorly framed, treating hip arthroscopy as a single procedure rather than a spectrum of interventions, which limits precision.
